# Relationship between Intrinsically Photosensitive Ganglion Cell Function and Circadian Regulation in Diabetic Retinopathy

**DOI:** 10.1038/s41598-020-58205-1

**Published:** 2020-01-31

**Authors:** Sirimon Reutrakul, Stephanie J. Crowley, Jason C. Park, Felix Y. Chau, Medha Priyadarshini, Erin C. Hanlon, Kirstie K. Danielson, Ben S. Gerber, Tracy Baynard, Jade J. Yeh, J. Jason McAnany

**Affiliations:** 1 Division of Endocrinology, Diabetes and Metabolism, Department of Medicine, University of Illinois at Chicago, Chicago, IL USA; 2 Biological Rhythms Research Laboratory, Department of Psychiatry & Behavioral Sciences, Rush University Medical Center, Chicago, IL USA; 3 Department of Ophthalmology and Visual Sciences, University of Illinois at Chicago, Chicago, IL USA; 4 Section of Adult and Pediatric Endocrinology, Department of Medicine, University of Chicago, Chicago, IL USA; 5 Division of Academic Internal Medicine and Geriatrics, Department of Medicine, University of Illinois at Chicago, Chicago, IL USA; 6 Department of Kinesiology and Nutrition, University of Illinois at Chicago, Chicago, IL USA

**Keywords:** Endocrinology, Endocrine system and metabolic diseases

## Abstract

Background: Intrinsically photosensitive retinal ganglion cells (ipRGCs) control non-visual light responses (e.g. pupillary light reflex and circadian entrainment). Patients with diabetic retinopathy (DR) show reduced ipRGC function, as inferred by abnormalities in the post illumination pupil response (PIPR). We explored whether ipRGC function in DR is associated with circadian outputs and sleep/wake behavior. Methods: Forty-five participants (15 without diabetes, 15 with type 2 diabetes (T2D) and no DR, 15 with T2D and DR) participated. ipRGC function was inferred from the PIPR (pupil size following stimulus offset). Circadian outputs were melatonin amplitude (overnight urinary 6-sulfatoxymelatonin (aMT6s)) and timing (dim light melatonin onset (DLMO)), and evening salivary cortisol levels. Sleep/wake patterns were measured with wrist actigraphy and insomnia symptoms were assessed subjectively. Results: Patients with T2D and DR had smaller PIPR and lower urinary aMT6s than other groups (p < 0.001). In adjusted regression models, smaller PIPR was associated with lower urinary aMT6s (β = 4.552, p = 0.005). Patients with DR were more likely to have no detectable DLMO (p = 0.049), higher evening salivary cortisol, greater insomnia symptoms and greater sleep variability compared to other groups. Sleep duration, efficiency and rest-activity rhythms were similar. Conclusion: Reduced ipRGC function in DR is associated with circadian dysregulation and sleep disturbances, although a causal relationship cannot be established in this cross-sectional study. Prospective mechanistic and intervention studies examining circadian and sleep health in these patients are warranted.

## Introduction

Diabetic retinopathy (DR) is a common diabetic complication, estimated to affect up to 30% of patients with diabetes^1^ with increasing prevalence^2^. DR is the leading cause of new cases of blindness in U.S. adults and costs ~$500 million annually^1^. Although DR is clinically defined by changes of the retinal vasculature, there is also mounting evidence showing that it is associated with early-stage dysfunction of the neural retina^3,4^. The loss of retinal ganglion cells (RGCs) is an important contributor to functional abnormalities in type 2 diabetes (T2D). In particular, the loss of melanopsin-expressing intrinsically photosensitive RGCs (ipRGCs)^5^ is thought to affect the control of pupil size, reducing the steady-state pupil size and altering the response of the pupil to light stimuli^6,7^. Previous studies show that the post illumination pupil response (PIPR), a measure of ipRGC function, is reduced in T2D patients with DR^8,9^. Likewise, retinal sections from six patients with severe DR revealed a significantly reduced density of ipRGCs^5^. These data suggested that DR is associated with ipRGC damage.

The ipRGCs are a crucial component of the entrainment of the human circadian system to the environmental light-dark cycle. By projecting directly into the central circadian clock, ipRGCs synchronize many physiological rhythms, including the secretion of melatonin, a neurohormone that influences sleep and glucose metabolism. Melatonin is secreted during the biological night by the pineal gland and is inhibited by light exposure^10^. ipRGC dysfunction in DR could lead to disturbed melatonin physiology and circadian dysregulation; however, this has not been examined. Chen and colleagues reported that nocturnal secretion of urinary 6-sulfatoxymelatonin (aMT6s), a melatonin metabolite, was low in T2D patients with proliferative DR compared to control participants, but there was no difference between non-proliferative DR and controls^11^. Similarly, nighttime plasma melatonin in patients with proliferative DR was found to be lower than controls, but no differences were found between those with non-proliferative DR and controls^12^. Another study also revealed lower serum melatonin secretion, especially at night, in patients with diabetes compared to controls, but the extent of DR was not reported in these individuals^13^. Our previous study revealed that overnight urinary aMT6s secretion was lower in patients with any degree of DR compared to those without DR^14^. More recently, Ba-Ali and colleagues reported that peak salivary melatonin at 4 a.m. was low in patients with diabetes compared to controls, but there were no differences between diabetes patients with and without DR^15^. They also reported that patients with DR had evidence of an abnormal rest-activity patterns suggestive of circadian misalignment^15^. Despite mixed results, these early studies suggested that diabetes and DR are associated with abnormal circadian/ melatonin regulation, but the link to ipRGC function has not been established.

Circadian dysregulation in DR could further adversely affect metabolic health in patients with T2D as the circadian system partly regulates the sleep/wake. Only two studies have assessed sleep duration and efficiency (a marker of sleep quality), in patients with and without DR, however, significant differences between groups were not found^14,15^. Furthermore, melatonin is known to affect glucose metabolism. We previously showed that nocturnal aMT6s was related to glycemic control in patients with diabetes^14^. In addition, there is clear evidence that sleep and circadian disturbances are associated with poor glycemic control in patients with diabetes^16,17^. These disturbances could potentially lead to a vicious metabolic cycle and exacerbate further diabetic complications in patients with DR.

The primary aim of this study was to examine whether ipRGC function is associated with circadian regulation and sleep behavior in patients with T2D and DR. ipRGC function was inferred by recording the PIPR (pupil size following stimulus offset). We assessed circadian regulation by measuring overnight urinary aMT6s which reflects melatonin amplitude, and melatonin timing as assessed by dim light melatonin onset (DLMO), along with evening cortisol profiles. Sleep was assessed by questionnaires and wrist actigraphy. We hypothesized that ipRGC dysfunction would be associated with disturbed melatonin physiology (e.g. low amplitude and/or abnormal timing) and sleep. Lastly, an exploratory analysis on the relationship between ipRGC function and glycemic control was performed.

## Results

Table 1 shows the characteristics of the 45 participants and comparisons among the three groups. Mean age, BMI and sex distribution were similar among groups. HbA1c levels tended to be higher in T2D with DR compared to T2D without DR, although this was not statistically significant.

**Table 1:** Characteristics of participants.

	Control (n = 15)	Diabetes without DR (n = 15)	Diabetes with moderate to severe DR (n = 15)	P value
Age (year)	52.4 (6.7)	53.7 (6.1)	56.8 (4.7)	0.116
Female	11	9	8	0.561
BMI (kg/m^2^)	34.3 (8.1)	32.9 (5.6)	30.0 (5.8)	0.204
Diabetes duration (yr)	N/A	9.5 (6.7)	17.3 (2.3)	0.110
HbA1c (%)	5.4 (5.1, 5.6)	7.8 (6.7, 10.0)	8.8 (8.2, 10.8)	0.059^d^
Relative PIPR	0.36 (0.25, 0.41)	0.29 (0.19, 0.40)	0.10 (0.02. 0.21)	<0.001^b,c^
***Melatonin variable***				
aMT6s/Cr ratio (ng/mg)	11.3 (6.6, 22.3)	15.5 (3.5, 29.6)	1.2 (0.4, 3.9)	<0.001^b,c^
DLMO presence	5/6	6/7	3/9	0.049
***Sleep variables***				
Insomnia severity index	2 (1,7)	4 (2, 8)	10 (7, 14)	0.001^b,c^
Sleep duration (min)	396 (39)	397 (69)	387 (73)	0.895
Sleep efficiency (%)	84 (80.87)	86 (79, 89)	81 (69, 86)	0.134
Sleep duration variability (min)	65.6 (27.6)	55.4 (21.9)	89.3 (42.8)	0.023^c^
Mid-sleep time (hh:mm)	02:54 (01:06)	3:18 (01:18)	03:51 (01:06)	0.104
***Rest-activity rhythm parameters***				
IV	0.78 (0.66, 0.84)	0.73 (0.57, 0.92)	0.77 (0.64, 0.88)	0.817
IS	0.48 (0.13)	0.43 (0.16)	0.45 (0.15)	0.608
M10-onset (h after midnight)	7.9 (6.1, 10.1)	8.1 (6.3, 10.7)	8.1 (6.9, 10.9)	0.694
L5-onset (h after noon)	11.8 (9.9, 12.2)	11.8 (11.1, 12.4)	12.7 (9.8, 13.7)	0.359
RA	0.88 (0.77, 0.93)	0.85 (0.75, 0.89)	0.77 (0.58, 0.85)	0.176
***Sleep disordered breathing assessment***				
AHI (events/h)*	7.9 (1.8, 10.8)	12.0 (4.8, 31.5)	16.2 (11.6, 28.8)	0.041^b^
***Neuropathy assessment***				
MNSI score	0 (0, 0)	1 (0, 3)	4.5 (1.5, 6.5)	<0.001^a,b,c^
The LF/HF ratio**	177 (54, 313)	183 (78, 397)	190 (94, 380)	0.953
RMSSD	29.0 (16.0, 66.0)	12.5 (5.8, 24.3)	9.5 (4.3. 31.5)	0.025^a,b^

^a^Significant differences between control vs T2D without DR; ^b^significant differences between control vs T2D with DR, ^c^significant differences between T2D without DR and T2D with DR.

^d^Comparison performed between T2D without DR and T2D with DR only.

*n = 15 for control participants, n = 14 for T2D without DR, and n = 12 for T2D with DR.

**n = 13 for control participants, n = 14 for T2D without DR, and n = 12 for T2D with DR.

AHI = apnea hypopnea index, BMI = body mass index, DLMO = dim light melatonin onset, IV = intradaily variability, IS = , interdaily stability, LF/HF ratio = low frequency/high-frequency ratio, MNSI = the Michigan Neuropathy Screening Instrument, RA = relative apmplitude, RMSSD = root mean square of the successive differences.

**Figure 1:**
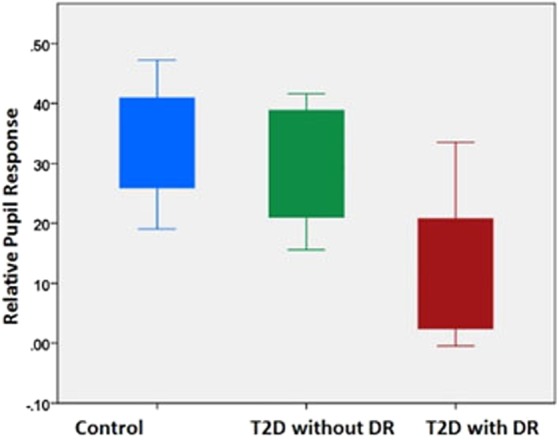
Comparison of relative pupil response (PIPR, median and interquartile range) among the three groups (p < 0.001); blue = control, green = T2D without DR, red = T2D with DR.

**Figure 2:**
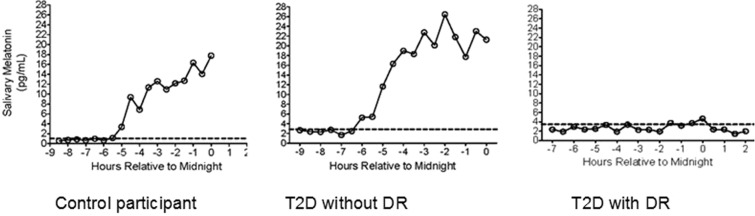
Evening salivary melatonin collected 7 h before to 2 h after average self-reported bedtime. Control participants (left) and participants with T2D without DR (middle) had a normal rise of evening melatonin before bedtime, thus detectable DLMO, while a participant with T2D and DR (right) had no detectable DLMO within the sampling time frame.

Pupillometry results showed that the T2D with DR group had significantly reduced ipRGC function as reflected by smaller relative PIPR amplitude compared to the other groups (p < 0.001) (Table 1 and Fig. 1). Patients with T2D and DR also produced less nocturnal aMT6s than the other groups (p < 0.001), and only 33% of patients with DR had detectable DLMOs during the evening sampling window compared to 83% in controls and 86% in patients with T2D and no DR (p = 0.049) (Table 1 and Fig. 2). Timing of DLMOs could not be compared among groups due to the small number of DR patients who had a detectable DLMO. When compared between control participants and T2D without DR, there were no significant differences in the timing of melatonin onset (p = 0.339).

Compared to controls and patients with T2D and no DR, T2D patients with DR had significantly greater insomnia symptoms, and increased night-to-night variability in sleep duration. Average sleep duration, sleep efficiency and mid-sleep time were not significantly different among groups. Sleep-disordered breathing (SDB) was more severe in T2D with DR, compared to T2D without DR.

For rest-activity rhythm variables, there were no significant differences among groups in intradaily variability (IV), interdaily stability (IS), M-10 onset or L5-onset. Relative amplitude was numerically lower in T2D with DR, although this was not statistically significant.

As expected, participants with T2D, with and without DR, had more signs of peripheral neuropathy as well as autonomic neuropathy as reflected by lower heart rate variability compared to controls. Sympathovagal balance was similar among groups.

### Associations between ipRGC function, urinary aMT6s and sleep

Correlations between demographic variables, PIPR, and nocturnal aMT6s are in Table 2. Lower PIPR amplitudes were significantly correlated with lower nocturnal aMT6s (p < 0.001). In addition, greater sleep duration variability, lower heart rate variability and peripheral neuropathy, were related to lower nocturnal aMT6s. Males had significantly lower nocturnal aMT6s than females. There was a trend for later mid-sleep times correlating with lower nocturnal aMT6s (p = 0.051). After adjusting for sex, peripheral neuropathy, and sleep duration variability, lower PIPR amplitudes remained significantly associated with lower nocturnal aMT6s (B = 4.552, p = 0.005), Table 3.

**Table 2:** Correlations between participants’ characteristics and relative PIPR and nocturnal aMT6s (N = 45).

	Relative PIPR		Urine aMT6s	
	r	p	r	p
Age	−0.199	0.191	-0.154	0.312
Male	−0.056	0.713	-0.332	0.028
BMI (kg/m^2^)	0.129	0.400	0.078	0.609
Relative PIPR			0.536	<0.001
aMT6s/Cr ratio	0.536	<0.001		
***Sleep variables***				
Insomnia severity index	−0.325	0.029	−0.250	0.097
Sleep duration (min)	0.019	0.904	−0.096	0.541
Sleep efficiency (%)	0.134	0.390	0.217	0.163
Sleep duration variability (min)	−0.142	0.364	−0.337	0.027
Mid-sleep time (hh:mm)	−0.136	0.384	−0.300	0.051
***Rest-activity rhythm parameters***				
IV	−0.056	0.720	0.025	0.874
IS	0.006	0.970	0.099	0.528
M10-onset (h after midnight)	−0.001	0.994	−0.053	0.734
L5-onset (h after noon)	0.048	0.759	−0.209	0.179
RA	0.142	0.364	0.180	0.247
***Sleep disordered breathing assessment***				
AHI	−0.281	0.079	−0.134	0.409
***Neuropathy assessment***				
MNSI score	−0.585	<0.001	−0.609	<0.001
The LF/HF ratio	−0.060	0.715	−0.276	0.089
RMSSD	0.298	0.066	0.414	0.009

AHI = apnea hypopnea index, BMI = body mass index, IV = intradaily variability, IS = , interdaily stability, LF/HF ratio = low frequency/high-frequency ratio, MNSI = the Michigan Neuropathy Screening Instrument, RA = relative apmplitude, RMSSD = root mean square of the successive differences.

**Table 3:** Multiple regression analysis with nocturnal aMT6s (ln) as an outcome.

Variables	B	SE	p
Intercept	1.839	0.637	
Relative PIPR	4.552	1.544	0.005
Male	−0.726	0.324	0.031
Sleep duration variability	−0.011	0.005	0.028
MNSI score	−0.164	0.092	0.081

B = unstandardized coefficient.

Lower PIPR amplitudes were also associated with greater insomnia symptoms, but aMT6s amplitude does not appear to be correlated with insomnia symptoms. Rather, lower urinary aMT6s was related to more variable sleep durations. There were no associations between the PIPR, urinary aMT6s and sleep duration, sleep efficiency or SDB severity.

### Association between ipRGC function and presence of DLMO

**Figure 3:**
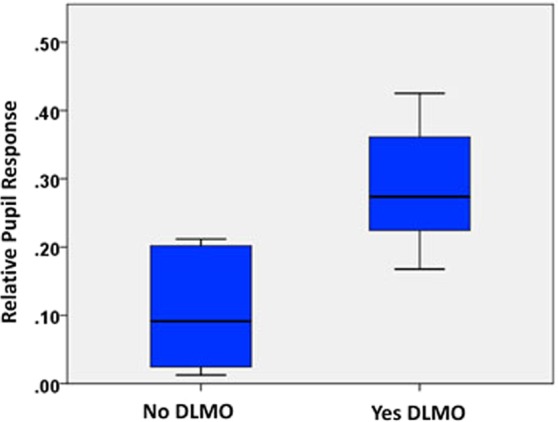
Comparison of relative PIPR between those with and without DLMO during the evening sampling timeframe (p < 0.001).

Figure 3 compares the median (interquartile range) PIPR amplitudes for participants with (n = 8) and without a detectable DLMO (n = 14). Those for whom a DLMO was not detected in the evening before bedtime had significantly smaller adjusted PIPR, suggesting lower ipRGC function (p < 0.001). As expected, compared to those with an evening DLMO, those without an evening DLMO had significantly lower urinary aMT6s [1.2 (0.5, 2.9) ng/mg vs. 5.7 (3.7, 13.5) mg/ml, p = 0.002)], along with more severe neuropathy (p = 0.013). In addition, sleep efficiency (p = 0.066) tended to be lower and sleep duration variability (p = 0.107) tended to be higher but these were not statistically significant. Sleep characteristics were otherwise comparable between groups.

### Evening salivary cortisol profile

**Figure 4:**
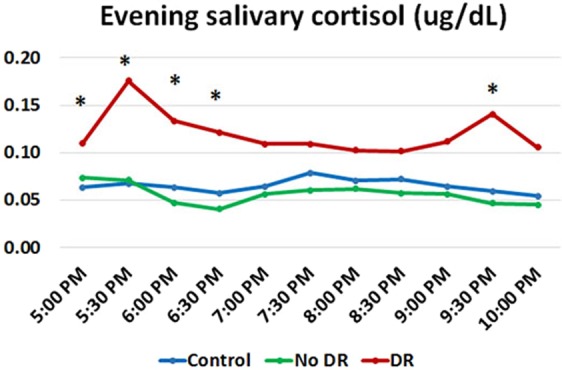
Evening salivary cortisol profile among the three groups of subjects (blue = control, green = DM without DR, red = DM with DR), *denotes p < 0.05.

Figure 4 shows evening salivary cortisol profiles (from 5:00–10:00 PM) among groups of participants. T2D with DR had significantly higher salivary cortisol at several time points than control participants and T2D without DR.

### Associations with glycemic control

Due to the small number of T2D participants overall, only exploratory bivariate analyses were performed between glycemic control and PIPR amplitude, sleep and urinary aMT6s. There were significant associations between poorer glycemic control (higher HbA1c) and lower PIPR amplitude (r = −0.425, p = 0.019), and higher sleep duration variability (r = 0.392, p = 0.039), but not urinary aMT6s (p = 0.157). No associations between HbA1c and other sleep variables were found.

## Discussion

This study confirmed previous findings of reduced PIPR amplitude in patients with type 2 diabetes with diabetic retinopathy^9^, indicating that these patients have abnormal ipRGC function. These data also demonstrated that circadian regulation is significantly disturbed in these patients as reflected by the absence of a detectable rise of melatonin before bedtime, low overnight urinary aMT6s levels, and elevated evening salivary cortisol. These results support the role of ipRGC function in circadian regulation of patients with diabetes, especially those with DR. Both PIPR amplitude and urinary aMT6s are associated with disturbed sleep (e.g. greater insomnia symptoms and sleep variability), which could be detrimental to the metabolic health of these patients and increase the risk for further complications. There are associations between PIPR amplitude and sleep variability with glycemic control but definite conclusions cannot be drawn due to the small number of participants with T2D. Collectively, these results give insight into the disturbances of circadian regulation and sleep in patients with DR, and their relationships with ipRGC function.

Increasing evidence demonstrates that patients with DR have low overnight melatonin secretion^11,12,15^. For the first time we now link melatonin dysregulation, including low overnight amplitude and absence of evening DLMO, to inferred ipRGC function in patients with DR. The lack of a detectable evening rise of melatonin in patients with DR is likely not due to acute photic suppression of melatonin because DLMO was measured in very dim light conditions. It is possible, however, that the DLMO could be detected at other times of the day in these patients but this will require a 24-hour assessment of melatonin to confirm. Interestingly, mid-sleep time – a behavioral correlate of DLMO phase in healthy adults^18^ – was similar in all three groups. It is possible that this temporal relationship between sleep behavior and circadian physiology does not hold in patients with DR since they showed similar mid-sleep times to the other groups despite the absence of an evening melatonin rise. ipRCG dysfunction can reduce the efficacy of photic entrainment of the central circadian clock, leading to circadian dysregulation, abnormal melatonin rhythm timing, and sleep disturbances. Ba-Ali and colleagues recently assessed circadian rhythm in DR, compared to controls and diabetes patients without DR, using diurnal salivary melatonin and cortisol pattern, and wrist activity rhythm^15^. The study revealed lower nocturnal and peak melatonin levels in patients with diabetes compared to controls, but there was no difference between those with and without DR^15^. Morning cortisol did not differ between groups. In contrast to our study, cortisol and melatonin were collected every 4 hours overnight while our study assessed evening levels every 30 minutes along with overnight urine collection, which may explain the differences in results. Ba-Ali and colleagues also showed that those with DR had increased variability in their activity-rest interval (intradaily variability, IV) as measured by actigraphy^15^, but this finding was not reproduced in our study. Cumulatively, these results suggest that there is some degree of circadian disturbance in patients with DR.

Disturbances in circadian regulation could lead to disturbed sleep and metabolic health. We found that patients with DR have increased night-to-night variability in their sleep pattern as reflected by sleep duration variability, which is associated with lower urinary aMT6s. Abnormal melatonin regulation could lead to weak circadian signaling and reduced ability to maintain a regular sleep schedule. Variability in sleep duration, likely associated with mild circadian misalignment, has been shown in several studies to be related to poorer glycemic control^19,20^. Experimental circadian misalignment in healthy volunteers in which sleep and eating times were shifted resulted in glucose intolerance, elevated blood pressure and inflammatory markers^21,22^. While the degree of sleep variability in DR patients seen in this study was much less than in the circadian experiments, our exploratory analysis suggested that there might be a relationship between sleep variability and glycemic control. We did not find a relationship between urinary aMT6s and glycemic control in this study. Nevertheless, it is well known that melatonin plays a role in glucose metabolism as its receptors are expressed in pancreatic β-cells, and it can modulate insulin secretion^23^. Low nighttime melatonin secretion was shown to be a predictor of insulin resistance and incident diabetes^24,25^. Elevated evening cortisol can also lead to increased insulin resistance^26^. These factors could further exacerbate glycemic control creating a vicious cycle leading to further complications in these patients. These findings should be confirmed in larger longitudinal studies.

The results of the current study, along with others^11,12,15^, raise the question of whether melatonin supplementation will be beneficial in patients with DR. Melatonin, both immediate release and long acting formulations, has been given before bedtime in patients with diabetes, up to 90 days, in a few randomized studies^27–30^. The results showed that melatonin could improve sleep quality^27,30^, reduce systemic inflammation^28^ and improve lipid profiles^28,31^, but the effects on glycemic control were mixed^27–30^, likely due in part to variations in treatment duration and background glycemic control. However, none of the studies have categorized the patients based on DR staging or measured circadian regulation. One melatonin agonist, tasemelteon, has been approved to specifically improve circadian regulation in patients with a non-24 hour sleep wake disorder^32^, but it has not been tested in DR. Whether patients with DR will improve sleep and circadian regulation, and possibly glycemic control, by melatonin supplementation remains to be explored.

The strength of this study is the detailed sleep and melatonin assessments. However, there are limitations. While we feel that the PIPR is largely ipRGC mediated and that subtracting responses elicited by photopically matched red and blue stimuli provides the most “pure” measure of melanopsin function possible under our conditions, it is possible that there are small contributions from other cell types that cannot be unequivocally dismissed. We did not assess circadian regulation across 24-hour cycle by gold standard methods (e.g. core body temperature or melatonin and cortisol sampling). The assessment of SDB was done by a portable monitor and not a gold standard polysomnography. While AHI was previously shown to be related to urinary aMT6s^14^, the result was not reproducible in this study, possibly due to different studied populations. There is a possibility that antidiabetic medications could influence biomarkers in this study, which we could not completely control for. However, when adjusting for insulin use in patients with diabetes only, PIPR was still a predictor of urinary aMT6s (results not shown). Lastly, the cross-sectional nature of the study could not elucidate the causal relationship between ipRGC dysfunction, DR and sleep and circadian disturbances. It is possible that the reduced ipRGC response could be a consequence of melatonin/circadian dysregulation, as ipRGCs are known to be under circadian control^33,34^. Besides ipRGC dysfunction, there is also a possible bidirectional relationship between the severity of diabetes and its comorbidities with sleep disturbances^35^. As patients with DR typically had more severe diabetes, they could be experiencing more nocturia, hypoglycemia or have more comorbidities leading to disrupted sleep.

In summary, we demonstrated that patients with DR exhibited characteristics of sleep and circadian disturbances including low amplitude of overnight melatonin, absence of DLMO, increased sleep variability and insomnia symptoms, and elevated evening cortisol levels. The melatonin dysregulation is strongly associated with ipRGC dysfunction, supporting the role of ipRGC function in circadian health of these patients. Whether these disturbances adversely affect metabolic health in patients with DR should be further investigated.

## Materials and Methods

### Participants

Adults with T2D without DR (n = 15), with moderate or severe non-proliferative DR (DR; n = 15), and participants without diabetes (control group; n = 15) aged 40-65 years, participated. Seven of these participants were a part of our previous study^9^. They were recruited by flyers and emails, and from the ophthalmology and endocrinology clinics at the University of Illinois at Chicago. Exclusion criteria included significant medical comorbidities (uncontrolled congestive heart failure, chronic obstructive pulmonary disease requiring oxygen, end stage renal disease or severe chronic liver disease such as cirrhosis, stroke, permanent pacemaker placement, and active psychiatric disease), performing night shift work, and use of exogenous melatonin, serotonin reuptake inhibitors, or illicit drugs.

Participants in the control group did not have a history of eye diseases or any history of previous ocular surgery, with the exception of uncomplicated cataract surgery. In addition, participants with a history of or presentation with, ischemic optic neuropathy, other optic nerve disease, glaucoma, other retinal vascular disorders such as retinal arterial occlusion, retinal vein occlusion, or ocular ischemic syndrome were excluded.

All participants provided written informed consent. The protocol was approved by the Institutional Review Boards at the University of Illinois at Chicago and Rush University Medical Center.

### The protocol

After informed consent was obtained, the participants completed an assessment of diabetes history (for T2D), insomnia symptoms and glycemic control. Dilated eye examination (if needed; see below) and pupillometry to assess ipRGC function were performed. The participants were then given an actiwatch to wear for one week, and had one night of home assessment of sleep-disordered breathing. At the end of the one-week period, they collected an overnight urine sample (for aMT6s) and completed a neuropathy assessment. For participants who completed the DLMO assessment, actigraphy recordings were obtained immediately before the assessment to appropriately time salivary melatonin sampling. The details of each procedure are outlined below.

### Assessment of diabetes history and glycemic control

Weight (kg) and height (cm) were measured. Body mass index (BMI) was calculated as weight (kg)/height (m)^2^. Research personnel interviewed participants with T2D about their diabetes history and management. Blood samples were obtained for Hemoglobin A1c (HbA1c) and assayed by Quest Diagnostics.

### Eye examination

Participants who had not had a complete eye exam within one year, or participants for whom the quality/interval of their previous eye examinations was deemed inadequate by the study’s ophthalmologist (F.Y.C.), underwent a comprehensive dilated eye examination. The examination included indirect ophthalmoscopy and slit lamp biomicroscopy. The stage of retinopathy in patients with diabetes was graded clinically based on the Early Treatment Diabetic Retinopathy Study (ETDRS) classification scheme^36^. The patients were grouped into no DR (ETDRS grade 10) and moderate-severe non-proliferative DR (ETDRS grade 43–53).

### ipRGC assessments

The pupillometry apparatus and methodology is described in detail elsewhere^9^. In brief, an LED-driven ganzfeld system was used for stimulus generation and display. Stimulus presentation and pupil recordings were performed monocularly using an infrared camera system with the other eye patched (usually the left). The infrared camera system measured the pupil diameter at a 60 Hz sampling frequency. Data were typically obtained from the right eye, but in rare cases in which the DR stage differed between eyes, the eye with the lower DR stage was tested. Test protocols intended to target the melanopsin (ipRGC) pathway were performed, as described in detail elsewhere^37,38^. In brief, subjects were dark adapted for 10 minutes and the PIPR was elicited using a short-wavelength (“blue;” dominant wavelength of 465 nm), high luminance (450 cd/m^2^) 1-sec flash presented in the dark. Responses to a minimum of two flashes, separated by at least 60 sec, were obtained and averaged for analysis. The pupil response to a photopically matched, 1-sec, long-wavelength flash (“red;” dominant wavelength of 642 nm) was also obtained from each subject. Pupillometry was in the morning around the same time for all participants.

Data obtained from pupillometry were analyzed using custom scripts programmed in MATLAB (MathWorks Inc., Natick, MA), which allowed for semi-automated analysis as described previously^37,38^. In brief, the response of the pupil to a flash of light (the pupillary light reflex; PLR) was normalized by the median steady-state (baseline) pupil size during the 1 sec preceding each stimulus onset. Normalization to baseline helped minimize the effects of inter-participant differences in the baseline pupil size. The sustained PIPR was measured as the difference between the normalized baseline and the median normalized PLR measured over a 5 to 7 sec time range following stimulus offset. Finally, the PIPR elicited by the red flash was subtracted from the PIPR elicited by the blue flash. All analyses discussed below were performed on this “relative” PIPR.

### Nocturnal urinary excretion of 6-sulfatoxymelatonin assessment

Participants were instructed to discard the last void prior to bedtime and collect each subsequent void until the first next morning void. The samples were stored in a dark bottle at room temperature, the total volume measured, and then stored at −80C until assayed. Urinary 6-sulfatoxymelatonin (aMT6s), a major melatonin metabolite, was measured by a competitive enzyme-linked immunosorbent assay (Bühlmann Laboratories AG, Schönenbuch, Switzerland). Urine creatinine was analyzed by Quest Diagnostics. Urinary aMT6s/creatinine ratios were calculated by dividing urinary aMT6s levels by urine creatinine concentration, expressed as ng/mg^11,14,24,25^. These values were referred to as nocturnal aMT6s.

### Dim-light melatonin onset (DLMO)

Participants completed a circadian phase assessment on one evening at the Biological Rhythms Research Laboratory at Rush University Medical Center to measure the dim light melatonin onset (DLMO), the most reliable marker of the central circadian clock in humans^39–41^. Twenty-two participants participated in this part of the protocol (6 controls, seven patients with T2D and no DR and 9 patients with T2D and DR). The phase assessments were timed based on participants’ unrestricted sleep schedules in the previous week. Saliva samples were collected every 30 minutes using Salivettes beginning 7 h before average bedtime and ending 2 h after average self-reported bedtimes. Participants remained seated and awake in comfortable recliners in dim light (<5 lux). The samples were immediately centrifuged and frozen, and later shipped on dry ice to SolidPhase, Inc (Portland, ME), where they were analyzed for melatonin using direct radioimmunoassay (RIA). The reported sensitivity of the assay is 0.7 pg/mL. Intra-assay and interassay variations are 12.1 and 13.2%. The DLMO threshold (in pg/mL) was defined based on the criteria of Voultsios *et al*.^42^. Sample times and melatonin values above and below the threshold were used to compute the DLMO (in clock time) using linear interpolation.

### Evening salivary cortisol profile

Saliva samples collected during DLMO assessments were assayed for cortisol levels using expanded range high sensitivity salivary cortisol enzyme immunoassay kit (item number:1-3002; Salimetrics, PA, USA). Salivary cortisol levels were shown to be well correlated to serum levels^43,44^. Samples thawed completely, were centrifuged for at 1500 × g for15 min and clear supernatant fractions were used in the assay. The intra- and inter assay variations were less than 10%. If values were missing, up to three values were interpolated using values at time point before and after.

### Sleep assessments

The Insomnia Severity Index is a 7-item scale that was used to assess insomnia symptoms^45^. Questions probed the severity of difficulties falling asleep, staying asleep, and problems with waking up too early in the past two weeks (response options ranged from none (0) to very severe (4)) and the impact of these difficulties on daily functioning, quality of life, and perceptions of their own sleep patterns (satisfaction, worry/distress). Total scores range from 0 to 28, with higher scores reflecting greater insomnia severity. A total score of 15 or higher indicates moderate to severe clinical insomnia.

Participants wore an Actiwatch spectrum wrist activity monitor (Philips Respironics, Bend, Oregon) for 7 days and completed a sleep log daily. The monitors use highly sensitive omnidirectional accelerometers to count the number of wrist movements in 30-s epochs. The software scores each 30-s epoch as sleep or wake based on a threshold of activity counts that is estimated using activity within the epoch being scored as well as the epochs 2 minutes before and after that epoch. Rest intervals to score sleep were defined by reported bed and wake-up times on daily sleep logs and if necessary, event markers from button presses at bedtime and wake-up time. Data were reviewed with participants when they returned the watch. Sleep duration was defined as the total amount of scored sleep at night, and sleep efficiency (a measure of sleep quality) was defined as percentage of time in bed spent sleeping. Both variables were calculated using Actiware 6.0 software, supplied by the manufacturer. Mid-sleep time was a midpoint between sleep onset and sleep end, both of which were calculated by the Actiware software. Additionally, standard deviation of sleep duration was used to reflect sleep variability. For each participant, the mean across all available nights was used. Data were available in 43 participants (15 controls, 14 T2D patients without DR, and 14 T2D patients with DR) with at least 6 days of actigraphy recording.

The presence and severity of sleep-disordered breathing (SDB), previously shown to be related to urinary aMT6s^14^, was assessed using an FDA-approved portable diagnostic device, WatchPAT 200 (Itamar Medical, Israel), which has been validated against polysomnography^46,47^. This non-invasive device is shaped similar to a large watch, worn on the non-dominant wrist immediately before bedtime and removed upon awakening in the morning. The device has two probes connecting to the participants’ fingers to measure changes in peripheral arterial tone (PAT) and oxygen saturation, along with a built-in actigraphy to measure sleep time. The severity of SDB is assessed by PAT Apnea Hypopnea Index (AHI) which is automatically generated by the software, using changes in the peripheral arterial tonometry. SDB is considered present if AHI ≥ 5. If the participants had a history of SDB and was using continuous positive airway pressure (CPAP) treatment, the assessment was performed while he/she was using CPAP.

### Rest-activity rhythm analysis

The rest-activity rhythm from actigraphy-derived wrist activity recordings was assessed by nonparametric variables^48^ using “nparACT” package for R as previously described^49^. We derived the following variables: intradaily variability (IV) which reflects the fragmentation of the rhythm, interdaily stability (IS) which quantifies the regularity of sleep patterns across days, M10-onset which is the start time of the most active 10-h period, L5-onset which is the start time of the least active 5-h period, and relative amplitude which takes into account the activity during the most active 10-h and the least active 5-h period, where a larger number reflects larger amplitude^48^. M10-onset was expressed as decimal hours after midnight (24:00), and L5-onset was expressed as decimal hours after noon (12:00).

### Neuropathy assessment

Signs of peripheral neuropathy, shown to be related to ipRGC function in T2D^50^, were assessed using the physician section of the Michigan Neuropathy Screening Instrument, MNSI^51^. This included assessments of foot deformities, dryness, ulceration, ankle reflexes, vibration perception and monofilament sensation. The possible total score is 10 with higher score reflecting more severe neuropathy.

Autonomic function assessments were performed by evaluating heart rate variability. These assessments were chosen as they pose minimal risk to participants with diabetes, and they largely reflect parasympathetic control which plays a significant role in pupillary response^52^. The *R-R intervals* were analyzed using HRV software (WnCPRS, Absolute Aliens Oy, Turku, Finland). In this analysis, R-R intervals were automatically detected and then visually inspected for accuracy and the occurrence of ectopic beats (premature, supraventricular, ventricular, atrial premature). They also are used to generate a tachogram of the R-R interval time event series. Both frequency and time domain analyses were performed. The two primary components of the frequency domain include the low-frequency (LF 0.04–0.15 Hz) and high-frequency (HF 0.15–0.40 Hz) spectra^53,54^. The LF/HF ratio is an indicator of sympathovagal balance^54^. From the time domain analyses, the primary HRV variable was the root mean square of the successive differences (RMSSD), which reflects parasympathetic modulation. All data acquisition and post-acquisition analyses were carried out in accordance with the standards put forth by Task Force of the European Society of Cardiology and North American Society of Pacing and Electrophysiology^55^.

### Statistical analysis

Data are presented as mean (SD), median (interquartile range, IQR) or frequency (%). Comparisons of variables among groups were performed with one way ANOVA, Kruskall-Wallis or Chi-square as appropriate. Post-hoc analyses were performed with Tukey or Mann Whitney U tests. Associations between variables were performed using Spearman’s correlations. To determine if ipRGC function (relative PIPR) was independently associated with urinary aMT6s (natural log transformed), multiple linear regression was performed adjusting for covariates. A possible interaction between ipRGC function and diabetes status on outcome urinary aMT6s was also examined; however, there was no significant interaction in the model thus no interaction terms were included.

Lastly, exploratory bivariate analyses were performed in only participants with diabetes to determine if ipRGC function, urinary aMT6s or sleep was associated with glycemic control (HbA1c). Analyses were performed using SPSS version 18 (Chicago, IL).
